# A novel role for atypical MAPK kinase ERK3 in regulating breast cancer cell morphology and migration

**DOI:** 10.1080/19336918.2015.1112485

**Published:** 2015-11-20

**Authors:** Rania Al-Mahdi, Nouf Babteen, Kiruthikah Thillai, Mark Holt, Bjarne Johansen, Hilde Ljones Wetting, Ole-Morten Seternes, Claire M Wells

**Affiliations:** 1 Department of Pharmacy; UiT The Arctic University of Norway; Tromsø, Norway; 2 Division of Cancer Studies; New Hunts House; Guy's Campus; King's College London; London, UK; 3 Randall Division for Cell and Molecular Biophysics and Cardiovascular Division; King's College London; London, UK

**Keywords:** actin filaments, cell adhesion, cell protrusion, cell motility, cell-cell adhesion, ERK3, mitogen-activated protein kinase 6

## Abstract

ERK3 is an atypical Mitogen-activated protein kinase (MAPK6). Despite the fact that the *Erk3* gene was originally identified in 1991, its function is still unknown. MK5 (MAP kinase- activated protein kinase 5) also called PRAK is the only known substrate for ERK3. Recently, it was found that group I p21 protein activated kinases (PAKs) are critical effectors of ERK3. PAKs link Rho family of GTPases to actin cytoskeletal dynamics and are known to be involved in the regulation of cell adhesion and migration. In this study we demonstrate that ERK3 protein levels are elevated as MDA-MB-231 breast cancer cells adhere to collagen I which is concomitant with changes in cellular morphology where cells become less well spread following nascent adhesion formation. During this early cellular adhesion event we observe that the cells retain protrusive activity while reducing overall cellular area. Interestingly exogenous expression of ERK3 delivers a comparable reduction in cell spread area, while depletion of ERK3 expression increases cell spread area. Importantly, we have detected a novel specific endogenous ERK3 localization at the cell periphery. Furthermore we find that ERK3 overexpressing cells exhibit a rounded morphology and increased cell migration speed. Surprisingly, exogenous expression of a kinase inactive mutant of ERK3 phenocopies ERK3 overexpression, suggesting a novel kinase independent function for ERK3. Taken together our data suggest that as cells initiate adhesion to matrix increasing levels of ERK3 at the cell periphery are required to orchestrate cell morphology changes which can then drive migratory behavior.

## Introduction

Cancer cell metastasis represents the greatest threat to cancer patient mortality. Cancer cell migration and adhesion are essential processes during metastatic spread. Cell migration depends on the coordinated regulation of dynamic rearrangements of the actin cytoskeleton accompanied by modifications in cell matrix adhesions which together drive the cellular shape changes that are observed during migration.^1,2^ Indeed cancer cells are reported to modify their shape and stiffness to interact with the surrounding tissue in order to migrate.^3^ Moreover, cell migration speed is controlled in part by the turnover rates of adhesion and dissociation between cells.^3^ It is well established that Rho family GTPases control cell migration and participate in the regulation of cancer metastasis. The Rho family GTPases, including Rho, Rac, and Cdc42 specifically regulate actin cytoskeletal dynamics and cell adhesion.^4,5^ and are known to induce morphological shape changes in cells.^6,7^ Cancer cells are thought to exhibit cellular plasticity whereby cell movement can be either individual (mesenchymal or rounded-amoeboid) or collective.^8^ In mesenchymal-type movement cells are more elongated^9,10^ and display Rac-driven actin-rich protrusions^9-11^ whereas in rounded-amoeboid movement, the cells have a rounded morphology with no obvious polarity. Here high levels of actomyosin contractility driven by Rho-ROCK and JAK-STAT3 facilitates elevated cells migratory speeds compared with elongated- mesenchymal cells.^9,10,12^ During metastasis cells are thought to be able to modify their shape (mesenchymal versus rounded) in response the physical barriers presented by the microenvironment. Indeed, formation of rounded-amoeboid cells can enhance tissue invasion, and its movement has been widely promoted as a tumor cell migration strategy.^13,14^ In vitro mesenchymal migration of recently plated cells have been described in two phases. Initially cells adhere and become elongated. Thereafter, the cell body tends to contract to generate traction force that leads to gradual forward gliding of the cell body. The speed generated by migration cycle is controlled by turnover rates of adhesion and dissociation between cells. In contrast, rounded-amoeboid cells change their shape by rapidly protruding and retracting extensions that have been described as pseudopods (false feet).^15^ and their movement results from alternating cycles of morphological expansion and contraction driven by cytoskeletal dynamics, shape change, and low cellular adhesion.^16^

The Rho family GTPases are known to deliver regulation of actin cytoskeletal dynamics via the interaction with downstream effectors in both normal and neoplastic cells.^17^ The group I p21 activated protein kinases (PAKs) are among the best-known effectors of Cdc42 and Rac1.^18-20^ Furthermore, PAKs are associated with tumor progression.^21^ Many potential targets have been identified for group one PAKs,^22-24^ but an interesting new substrate for mammalian PAK1–3 is the atypical MAP kinase ERK3.^25,26^ ERK3 together with ERK4 are considered to be atypical members of the MAP kinase family. This is due to the fact that both proteins lack the canonical Thr-X-Tyr motif found in the activation loop of the classical MAP kinases and they have a unique C-terminal extension. The activation loop of ERK3 and ERK4 consists of a Ser-Gly-Glu with Ser as a single phosphorylation site. This serine in ERK3 is constitutively phosphorylated by the group one PAKs in resting cells and its phosphorylation is not changed in response to extracellular stimuli that activates the classical MAP kinases.^25,26^ ERK4 is a relatively stable protein, while ERK3 is considered as non-stable protein and is rapidly degraded by the ubiquitin proteasome pathway.^27,28^ To date the physiological function of ERK3 is unclear. However, genetic ablation of the *Erk3* gene has revealed that ERK3 plays an important role in fetal growth and lung maturation.^29^ The only identified ERK3 substrate is MAPK-activated protein kinase-5 (MK5 or PRAK).^30^ MK5 was demonstrated not only to act as a substrate for ERK3, but activated MK5 is also able to phosphorylate ERK3 both in vitro and in vivo,^30^ indeed the interaction between ERK3 and MK5 regulates the stability of ERK3.^30^ Several experimental studies has shown that MK5 is involved in a wide range of biological processes including cytoskeletal rearrangement by F-actin remodeling^31-33^ and tumor suppression.^34^ However, a role for ERK3 in cell adhesion and/or migration has not been investigated.

In this study we demonstrate that ERK3 protein levels are elevated as MDA-MB-231 breast cancer cells adhere to collagen I, which is concomitant with changes in cellular morphology where cells become less well spread following nascent adhesion formation. We further show that exogenous expression of ERK3 delivers a comparable reduction in cell spread area, while depletion of ERK3 expression increases cell spread area. Furthermore, we find that ERK3 overexpressing cells exhibit an increased cell migration speed. Surprisingly, exogenous expression of a kinase inactive mutant of ERK3 phenocopies ERK3 overexpression suggesting a novel kinase independent function for ERK3. Taken together our data suggest that as cells initiate adhesion to matrix, increasing levels of ERK3 at the cell periphery are required to drive cell morphology changes which can then drive migratory behavior.

## Results

### MDA-MB-231 cells show a significant decrease in spread area following nascent adhesion

**Figure 1.**
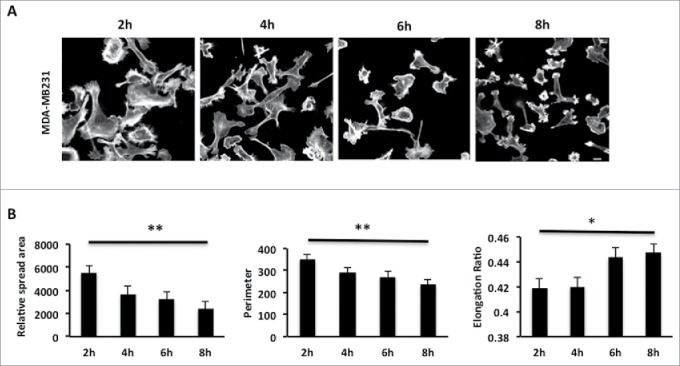
MDA-MB-231 cells show a significant decrease of relative spread area after 8 hours of seeding. (**A**) MDA-MB-231 cells were seeded onto collagen I coverslips for the following time course 2, 4, 6, 8 hours and were fixed and stained with TRITC-phalloidin to show F-actin and Dapi. Images were taken by confocal microscopy. (**B**) Cell spread area, perimeter and elongation ratio were calculated using ImageJ (NIH) software. The results shown are mean ± s.e.m of over 30 cells from each population in each of three separate experiments. Statistical significance was analyzed using the student test, *P ≤ 0 .05 and **P ≤ 0 .005. Scale bar: 10 μm.

**Figure 2.**
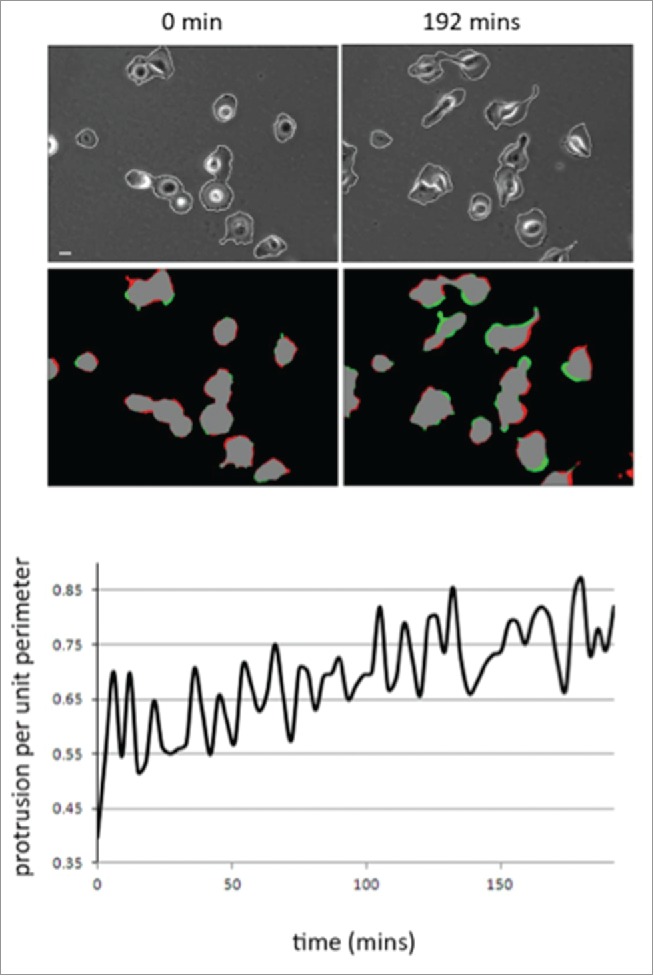
Cell protrusion activity increases as MDA-MB-231 cells adhere to collagen. (**A**) Representative images of movie stills (t=0 and t=192 mins) from a movie of MDA-MB-231 imaged following plating on collagen I. Phase contrast to reveal cell outline detection (see materials and methods) and red/green pseudocolour to reveal areas of protrusion (green) and retraction (red). (**B**) Quantification of mean protrusion per unit perimeter over time. N=2 movies (20 cells). Images were quantified using in house Mathematica^TM^ software.

The MDA-MB-231 breast cancer cell line is routinely used to study adhesion, migration and invasion events. However, we found that the morphological response of MDA-MB-231 cells following initial adhesion to collagen I has not been previously characterized. To explore the morphological response of MDA-MB-231 cells we fixed and stained cells plated on collagen I for up to 8 hours (**Fig. 1**). Cell shape analysis revealed that as cells are forming nascent adhesions the cell perimeter and spread area significantly decreases but concomitantly the cell becomes more polarized (as revealed by the elongation ratio). We were surprised to find that cells exhibited a reduced cell area following plating and wondered whether this was reflected by a lack of protrusive activity in these cells. To test protrusive activity we made time-lapse movies of cells immediately following plating on collagen I. Using in-house software specifically designed to measure protrusive activity over time we were able to ascertain that despite the reduction in spread area all cells exhibit protrusive activity –indeed the rate of protrusive activity increases over time (**Fig. 2**). Thus the cells are exhibiting dynamic changes in the actin cytoskeleton as well as increased levels of contractility as nascent adhesions are replaced by more mature migratory adhesions.^35^

### ERK3 protein levels are positively correlated with morphological changes following adhesion

**Figure 3.**
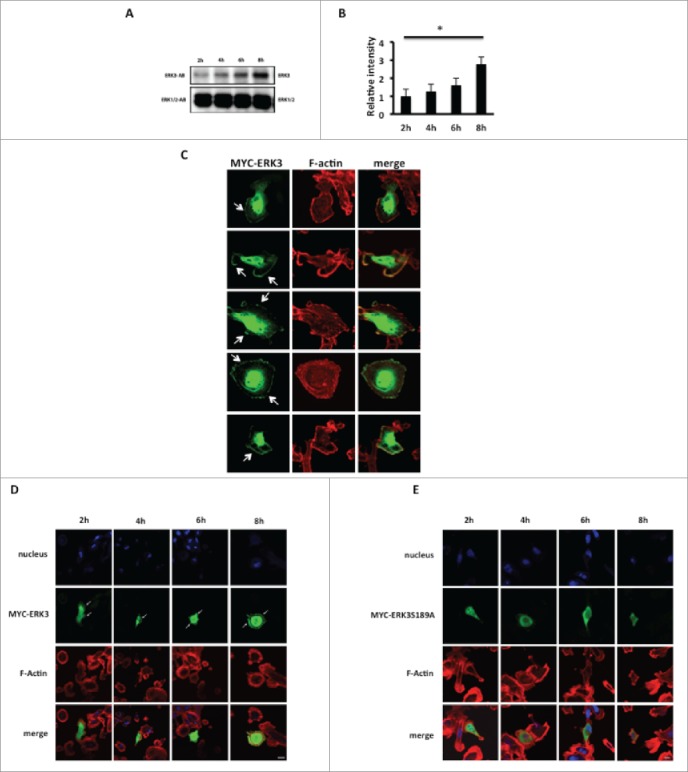
ERK3 increasing level is coincided with the significant decrease of relative spread area. (**A**) MDA-MB-231 cells were seeded onto collagen I coated six-well plate and harvested at the following time course 2, 4, 6, 8 hours and probe for endogenous ERK3. The figure shown is a representative of three separate experiments. (**B**) Relative intensity was calculated for the time course blot 2, 4, 6, 8 hours and analyzed using student test. The figure shown is a representative of three separate experiments. (**C**) Overexpressed ERK3 localizes mainly in the nucleus and at the plasma membrane of the cell. MDA-MB-231 cells were transfected with MYC-ERK3 for 24 hours, fixed and stained with TRITC-phalloidin for F-actin (red) and MYC tag as required (green). (**D**) MDA-MB-231 cells were transfected with MYC-ERK3 or (**E**) MYC-ERK3S189A for 24 hours. The transfected cells were then seeded onto collagen I plates for the following time course 2, 4, 6, 8 hours and were fixed and stained with TRITC-phalloidin to show F-actin and Dapi. For ERK3 detection (green), ERK3 monoclonal antibody was used followed by Alexa Flour 488 anti-mouse. Confocal images were taken. Scale bar: 10 μm.

Several recent studies.^25,26,36^ have suggested a function for ERK3 in cytoskeletal dynamics. This suggestion comes from the discovery of ERK3 as a substrate for the group one p21 activated kinases, which have been implicated in cell spreading^37^ and the observation that ERK3 can regulate the expression of matrix metalloproteinases MMP2, 9 and 10 through phosphorylation of the transcriptional co-activator SRC-3.^25,26,36^ The biological activity of ERK3 is thought to be regulated through its cellular abundance.^28^ Thus, having identified that there is a dramatic shift in cell morphology within the first 8 hours of cell plating we proceeded to analyze the level of ERK3 expression over this time frame. MDA-MB-231 were seeded on collagen I and lysed at appropriate time points as dictated by our spreading analysis (**Fig. 1**). We find that ERK3 levels are significantly increased during the adhesion, spread area reduction and polarization phase (**Fig. 3A**-**B**). Thus suggesting a direct correlation between ERK3 expression levels and changes in cellular morphology, where ERK3 expression levels were highest in the more rounded cells, which had been adhered to the substratum for 8 hours. Moreover, increased ERK3 protein levels following 8 hours of cell seeding were independently reproducible in a second cell line (**Fig. S1A**). In agreement with previous publication^38^ we were able to confirm that the increase in ERK3 expression is post-translational.(**Fig. S1B**).

### ERK3 is localized at the cell periphery

Our data (**Figs. 1 and 2**) suggest that ERK3 may influence cellular morphology. We therefore sought to determine the subcellular distribution of ERK3 in our cells. MYC-tagged ERK3 was transiently expressed in MDA-MB-231 cells and the subcellular localization observed by confocal microscopy. As expected a significant proportion of MYC-ERK3 was distributed in the nucleus,^27,39,40^ however we were also able to detect MYC-ERK3 at the periphery of the cells (**Fig. 3C**). We confirmed the peripheral localization of ERK by also detecting GFP-tagged ERK3 at the cell periphery (**Fig. S1C**) and ultimately by detecting endogenous ERK protein localized at the cell periphery (**Fig. S1D**). Thus confirming that overexpression had not influenced the ERK3 localization. Taken together these data demonstrate that a significant proportion of ERK3 is localized in the periphery of the cells at the plasma membrane. Given our finding that ERK3 expression levels are positively correlated with substratum adhesion we also sought to define the localization of ERK3 during nascent adhesion. Interestingly while MYC-ERK3 can be clearly detected at the cell periphery following 8 hours adhesion (**Fig. 3D**), a mutant ERK3 (MYC-ERK3S189A) which cannot be phosphorylated by PAK1 cannot be detected.(**Fig. 3E**).

### Exogenous expression of ERK3 can induce morphological changes

**Figure 4.**
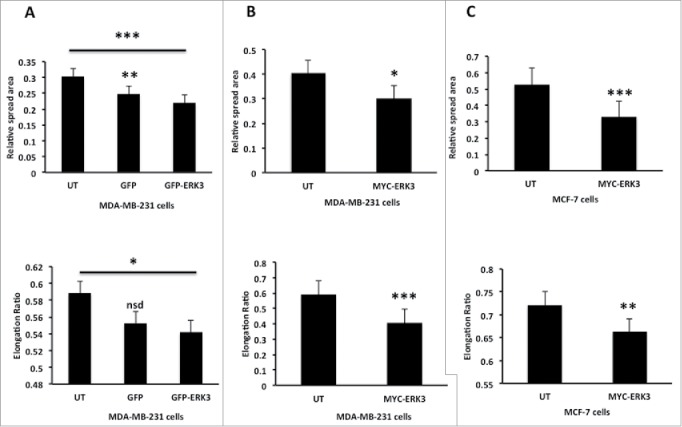
Over expression of ERK3 in different breast cell lines induces a reduction in spread area and elongation ratio. (**A**) MDA-MB-231 cells were transfected with GFP control vector or GFP-ERK3 for 24 hours, the cells were than fixed and stained with TRITC-phalloidin for F-actin and Dapi. (**B**) MDA-MB-231 Cells were transfected with MYC-ERK3 for 24 hours, the cells were than fixed and stained with TRITC-phalloidin for F-actin, Dapi and MYC tag as required. (**C**) MCF-7 cells were transfected with MYC-ERK3 for 24 hours, the cells were than fixed and stained with TRITC-phalloidin for F-actin, Dapi and MYC tag as required. All relative spread area and elongation ratio were calculated using ImageJ (NIH) software. The results shown are mean ± s.e.m of over 30 cells from each population in each of three separated experiments. Statistical significance values were calculated using Student's t-test, *P ≤ 0.05, **P ≤ 0 .005 and ***P ≤ 0.0005.

Given that ERK3 protein levels are modulated as cells undergo morphological changes and ERK3 is localized at the cell periphery, we reasoned that ERK3 might play a role in regulating cell shape changes. To test this hypothesis we overexpressed either GFP-ERK3 or MYC-ERK3 in MDA-MB-231 cells and tested the impact on cell spread area and shape. Interestingly cells overexpressing ERK3 regardless of the tag induced a reduction in cell spread area. Furthermore, exogenous expression of ERK3 (likely to be higher than levels generated by collagen adhesion) also induced cell rounding (**Fig. 4A**-**B**), a morphology that was not induced by exposure to transfection reagent alone (**Fig. S1E**). Moreover, the impact of ERK3 overexpression could also be detected in a second breast cancer cell line; MCF-7 where a reduction in cell spread area and cell elongation was also detected (**Fig. 4C**). Thus ERK3 is able to drive morphology changes in cells.

### ERK3 overexpression drives increased cell migration speed

**Figure 5.**
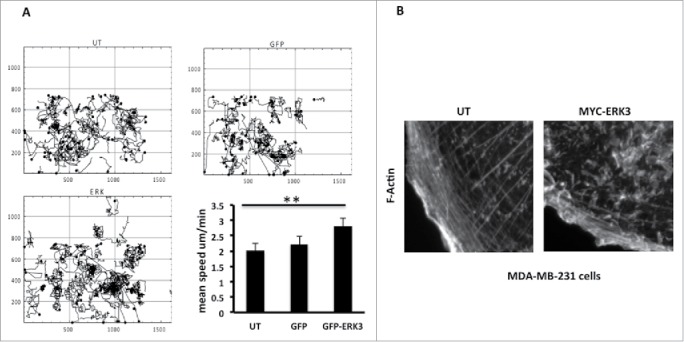
ERK3 has induced the MDA-MB-231 cells mobility and the actin cytoskeleton rearrangement. (**A**) MDA-MB-231 cells were transfected with GFP control vector or GFP-ERK3 for 24 hours. Cell images were collected using a Sensicam (PCO Cook) CCD camera, taking a frame every 5 minutes for 16 hours from each of the six wells using AQM acquisition software. Subsequently, cells were tracked using AQM tracker. Over 10 cells were tracked over six separate films from three separate experiments for each experimental condition. Mathematical analysis was then carried out using Mathematica 6.0™ workbooks (ANOVA). Mean track speeds for each condition were compared using the Student's T-test, **P ≤ 0 .005. (**B**) MDA-MB-231 cells were transfected with MYC-ERK3 for 24 hours, the cells were than fixed and stained with TRITC-phalloidin for F-actin, Dapi and MYC tag as required. Images of F-Actin were taken using Time-lapse microscopy. An increase of ERK3 level has an effect in F-Actin organization.

Cell morphology changes are intrinsically linked to cell migration potential as F-actin rearrangement, cell adhesion modification and cell polarization are all processes associated with cellular motility.^1^ Given that overexpression of ERK3 induces a reduced spread area and decreases cell elongation we speculated that these cells may have an increased migratory speed, as has been suggested in melanoma.^12^ We have used Time-lapse microscopy to observe and track the movement of the GFP-ERK3 transfected cells. Tracking analysis of GFP-ERK3 transfected cells revealed that ERK3 overexpressing cells exhibit an increase in mean cell migration speed compared to control cells (**Fig. 5A**). The GFP-ERK3 cells were moving with a mean cell speed of ± s.e.m. = 2.822±0.2213 μm/min, un-transfected cells ± s.e.m. = 2.012±0.1558 μm/min and GFP transfected cells ± s.e.m. 227±0.2187 μm/min. Changes in cell morphology and cell migration speed could be driven by changes in actin cytoskeletal dynamics. We therefore made a detailed examination of the F-actin rearrangement in control and overexpressing cells. We did indeed find that ERK3 overexpressing cells lost prominent actin stress fibers at the cell periphery and displayed an increase in peripheral ruffling (**Fig. 5B**) which was not induced by exposure to transfection reagent alone.(**Fig. S1F**).

### ERK3 overexpression induces cell scattering

**Figure 6.**
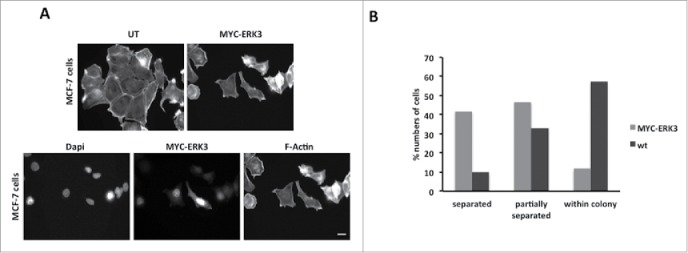
ERK3 transfected cells tend to dissociate from neighboring cells. (**A**) MCF-7 cells were transfected with MYC-ERK3 for 24 hours, the cells were then fixed and stained with TRITC-phalloidin for F-actin, Dapi and MYC tag as required. ERK3 transfected cells lose their contact with other cells. (**B**) The percentage of MCF-7 cells that had been detected to be dissociated. 41.7% of cells were totally isolated, 46.6% cells were partially separated and 11.6% were within the colony. Scale bar: 10 μm.

We have already demonstrated that exogenous expression of ERK3 induces the same morphological changes in MDA-MB-231 and MCF-7 cells (**Fig. 4C**). We therefore proceeded to test if overexpression of ERK3 could also promote migration in MCF-7 cells. A routine measure of MCF-7 motility is the cell scattering assay.^41^ Immunofluorescence observations of MYC-ERK3 expressing cells suggested that these cells were more likely to be distant from the cell colony (separated cells) (**Fig. 6A**). We therefore scored control and MYC-ERK3 expressing cells for the cell scattering event (cells have moved away from the colony and are complete separate). Our quantification revealed that while only 9.8% of wild-type cells were classified as separated 41.7% of MYC-ERK3 cells were separated from the cell colonies (**Fig. 6B**). Thus exogenous expression of ERK3 promoted cell migration in a second cell line.

### Depletion of ERK3 results in an increase in cell spread area

**Figure 7.**
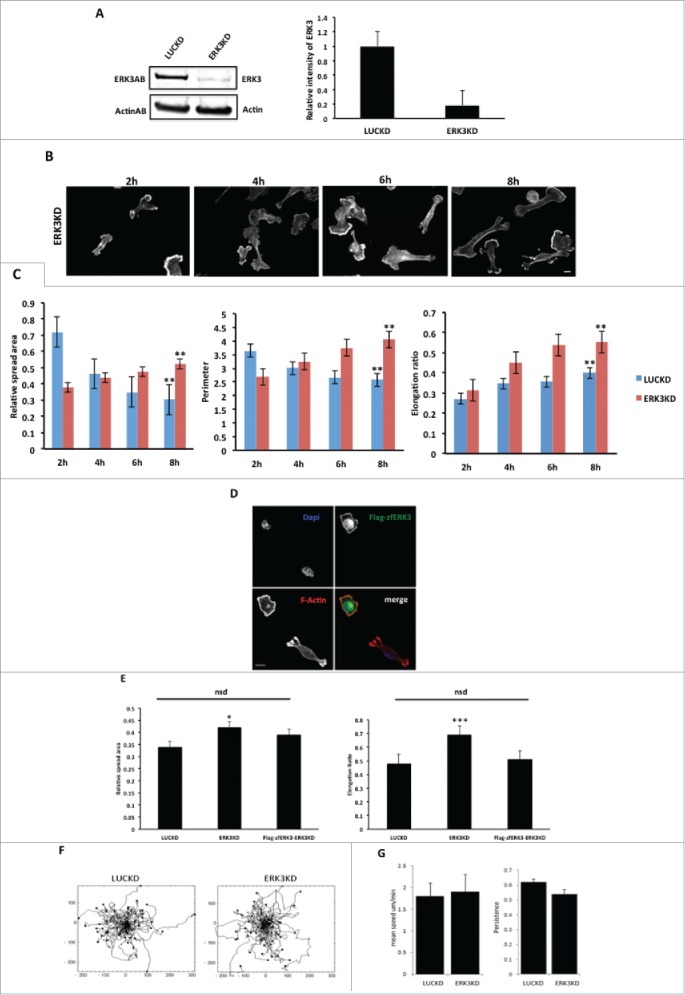
The depletion of ERK3 protein induces an increasing in spread area and elongation ratio. (**A**) ERK3 was knocked down in MDA-MB-231 cells. For control LUC protein was knocked down (see material and methods) (**B**) MDA-MB-231 cells were seeded onto collagen I coverslips for the following time course 2, 4, 6, 8 hours and were fixed and stained with TRITC-phalloidin to show F-actin and Dapi. Cells were imaged by Time-lapse microscopy. (**C**) Cell spread area, perimeter and elongation ratio were calculated using ImageJ (NIH) software. The results shown are mean ± s.e.m of over 30 cells from each population in three separate experiments. Statistical significance was analyzed using the student test, *P ≤ 0 .05 and **P ≤ 0 .005. Scale bar: 10 μm. (**D**) LUCKD, ERK3KD and ERK3KD cells transfected to express Flag-zfERK3 (Flag-zfERK3-ERK3KD) were seeded onto collagen I coverslips for 8 hours, fixed and stained with TRITC-phalloidin to show F-actin, Dapi and Flag tag as required. (**E**) Cell spread area and elongation ratio of LUCKD, ERK3KD and Flag-zfERK3-ERK3KD were calculated using ImageJ (NIH) software. The results shown are mean ± s.e.m of over 30 cells from each population in three separate experiments. Statistical significance was analyzed using the student test, *P ≤ 0 .05 and ***P ≤ 0 .0005. Scale bar: 10μm. (**F**) LUCKD and ERK3KD cells were seeded on collagen I wells and cell images collected for 16 hours using AQM acquisition software. Cell track plots for LUCKD and ERK3KD cells with all tracked plotted from 0,0 are illustrated (**G**) Individual cells were tracked and the mean migration speed and persistence of direction was calculated using in house Mathematica^TM^ software.

Our data strongly suggest that ERK3 can mediate changes in cellular morphology. Given that ERK3 expression increases following cell plating on collagen I we next tested whether the cell shape changes we had observed (**Fig. 1**) could be maintained in cells depleted of ERK3 expression. We therefore generated MDA-MB-231 cells stably depleted of ERK3 expression (**Fig. 7A**). Control (LUCKD) and ERK3 depleted cells (ERK3KD) were seeded onto collagen I over an 8 hours time course as previously described (**Fig. 1**). Cells were stained for F-actin (**Fig. 7B**) and cell images quantified for cell spread area and elongation as before (**Fig. 1B** and **Fig. 7C**). We find that control cells exhibit a reduction in cell spread area over time concomitant with an increase in cell elongation. In contrast ERK3KD cells initially exhibit a reduced spread area but over time continue to spread and by 6h have an increased spread area compared to control cells. Thus suggesting that ERK3 is required to deliver reduced spread area 8h post plating. Interestingly, both control and ERK3 depleted cells elongate over time, indeed ERK3KD cells have a significant increase in elongation ratio over control cells at 8h post plating (**Fig. 7C**). These observations suggest that ERK3 expression is not only required to reduce spread area but also to prevent hyper-elongation of the cells. To further validate these observations a siRNA rescue experiment was performed using a cross species^42-44^ rescue construct (zfERK3) that would not be targeted by shRNAi sequences present in the ERK3KD cells. Importantly we first established that overexpression of zfERK3 induces cell rounding as described for HuERK3 (**Fig. S1G** and **Fig. 4**) thus demonstrating it is functional in MDA-MB-231 cells. Subsequently, we found that siRNA resistant zfERK3 expression in ERK3KD cells abrogated the hyper-elongation exhibited by ERK3KD cells and reduced the spread area to control levels (**Fig. 7D**-**E**). Indeed, zfERK3 was also clearly localized to the cell periphery following nascent adhesion formation (**Fig. 7D**). Having established that overexpression of ERK3 can induce cell rounding and increased mean cell migration speed we next tested whether depletion of ERK3 influenced migratory behavior. In contrast to overexpression of ERK3, cells depleted of ERK3 exhibited a mean migration speed that was not significantly different from control cells (**Fig. 7F**-**G**). Thus suggesting that the enhanced elongation of ERK3KD cells observed in (**Fig. 7C**) does not impede cell migration.

### ERK3 driven morphological changes are not kinase dependent

**Figure 8.**
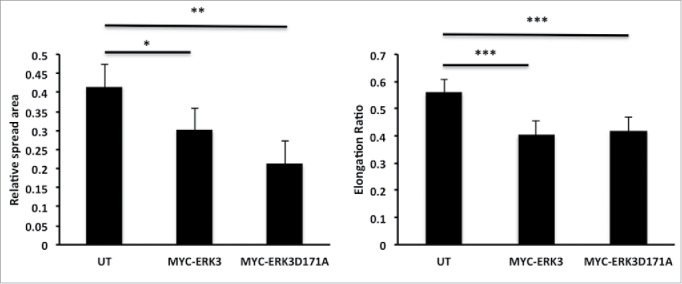
ERK3 kinase activity has no effect on MDA-MB-231 cell morphology alteration. MDA-MB-231 cells were transfected with either MYC-ERK3 or a Kinase dead mutant MYC-ERK3 (MYC-ERK3D171A). After 24 hours, the cells were fixed and stained with TRITC-phalloidin for F-actin, Dapi and MYC tag as required. Cell spread area and elongation ratio were calculated using ImageJ (NIH) software. The results shown are mean ± s.e.m of over 30 cells from each population in each of three separate experiments. Statistical significance was analyzed using the student test, *P ≤ 0 .05 and **P ≤ 0 .005 and ***P ≤ 0 .0005.

Our data suggest that ERK3 can drive cell morphology changes that translate into changes in cell migration potential (**Fig. 4 and 5**). ERK3 is an atypical Mitogen-activated protein kinases (MAPK6) and its only known physiological function is to phosphorylate and activate MK5.^30^ It has been previously established that a kinase dead mutant MYC-ERK3D171A is incapable of MK5 activation.^30,45^ We therefore proceeded to test the requirement for ERK3 kinase activity during ERK3-induced MDA-MB-231 cell morphological changes. MDA-MB-231 cells were transfected with kinase-dead mutant MYC-ERK3D171A and the spread area and elongation ratio were calculated using ImageJ. We were surprised to discover that overexpression of MYC-ERK3D171A also induced a significant reduction in cell spread area indeed greater than that seen with overexpression of MYC-ERK3 (**Fig. 8**). Moreover, exogenous expression of MYC- ERK3D171A also reduced cell elongation to a comparable level with exogenous expression of MYC-ERK3 (**Fig. 8**). These data suggest that ERK3 mediated morphological changes are not mediated via phosphorylation of MK5 or any other as yet unidentified substrate.

## Discussion

Very little is known about the physiological role of the atypical MAP kinase ERK3. In this study we identify specific morphological changes in cell spread area and cell shape as MDA-MB-231 breast cancer cells adhere to a collagen I substratum. Whereby cells become less well spread following nascent adhesion formation but exhibit enhanced elongation. We proceed to correlate these morphological changes with an increase in ERK3 protein levels and further show that exogenous expression of ERK3 delivers a comparable reduction in cell spread area, while depletion of ERK3 expression increases cell spread area. In addition, we find that rounded ERK3 overexpressing cells exhibit an increased cell migration speed. The unexpected observation that overexpression of a kinase deficient ERK3 mutant phenocopies wild-type ERK3 suggests a novel kinase independent function for ERK3. Taken together our data suggest that as cells initiate adhesion to matrix increasing levels of ERK3 at the cell periphery are required to drive cell morphology changes which can then drive migratory behavior.

Our detailed analysis of MDA-MB-231 cells plated onto collagen I has revealed that the cells exhibit a morphology plasticity that ultimately leads to smaller more polarized cells with high levels of protrusive activity. We would hypothesize that this behavior renders the cells able to begin efficient 2D migration. We have found that ERK3 is playing a role in this initial modulation of cell morphology where cells with a loss of ERK3 expression are unable to adopt the same morphological shape as control cells. In our studies ERK3 is required to protect the cell from hyper-elongation and we would speculate that this requirement is attributable to a role for ERK3 in cell contractility; a hypothesis supported by the changes in cell shape and migration observed in ERK3 overexpressing cells as discussed below.

Several recent studies have suggested a function for ERK3 in cell migration.^25,26,32,36^ This suggestion comes from the discovery of ERK3 as a substrate for the group one p21 activated kinases and the observation that ERK3 can regulate the expression of matrix metalloproteinases MMP2, 9 and 10 through phosphorylation of the transcriptional co-activator SRC-3.^25,26,36^ ERK3 protein is known to have a short half-life compared to other MAPKs and its biological activity is thought to be regulated through its cellular abundance.^28^ Our data suggests the cells specifically increase ERK3 protein levels during early phase cell adhesion and polarization an increase that was reproducible across cell lines. Indeed, a similar increase in ERK3 protein expression has been observed in all of the cells lines we have tested and is also observed (although at lower level) when cells are plated on fibronectin or ordinary plastic tissue culture dishes (data not shown). Moreover, Crowe *et al* also observed that plating of squamous cell carcinoma cells onto collagen IV gave rise to increase in ERK3 protein expression.^38^

Interestingly we find that this rise in endogenous ERK3 protein level coincidences with a decrease in the cell spread area, perimeter and increase in the cell elongation ratio. The rise in ERK3 levels is likely to be due to an increase in ERK3 stability that is induced upon cell plating. The source of this increased stability is not yet known but could involve re-localization of ERK3 to the periphery and/or phosphorylation via PAK. In contrast it could also involve an as yet unidentified interaction partner that protects ERK3 from ubiquitination^28^ following cell plating.

Our work points to a role for ERK3 in cell contractility, mediating constraint of the cell periphery. While one study has suggested a ERK3 localization in the Golgi/ER-Golgi intermediate compartment (ERGIC).^46^ many studies have reported that ERK3 is constitutively localized into the cytoplasmic and nuclear compartments.^28,39^ However none have described a peripheral localization close to the plasma membrane that might be required for driving cell contractility. The cytoplasmic distribution of ERK3 is known to be dependent on nuclear export and the nucleoplasmatic shuttling of ERK3 is required for some of its biological functions.^39^ Co-expression of ERK3 with its physiological partner MK5 results in re-localization of both proteins in the cytoplasm. This re-localization of ERK3 and MK5 is dependent on a direct protein-protein interaction between the two proteins via ERK3 phosphorylation at serine 189. The only known kinases that are able to phosphorylate ERK3 at serine 189 are the group one PAK^25,26^ and inhibition of group one PAK results in nuclear accumulation of ERK3.^25^ However, the subcellular distribution of ERK3 has so far almost exclusively been studied using ectopically expressed ERK3.^39,40,47^ Using a monoclonal antibody we were able to detect endogenous ERK3 both in the nucleus and in the cytoplasm, but more importantly we were also able to detect a specific localization of ERK3 in the periphery close to the cell membrane. Similar localization was also observed with ectopically expressed ERK3. This localization of ERK3 close to the cell membrane is compatible with a function for the kinase in cell spreading and migration. Indeed, we were able to show a distinct peripheral localization of ERK3 at the cell periphery following nascent adhesion formation that required phosphorylation at serine 189. However, the interaction partners for ERK3 in the cell periphery remain to be elucidated. Due to technical limitations it is not possible to image endogenous localization of MK5. Although, a recent study identified MK5 as an *in situ* substrate for focal adhesion kinase (FAK).^48^ Interestingly MK5 is tyrosine phosphorylated during cell adhesion and this phosphorylation induces localization at focal adhesions.^48^ However, localization of MK5 to the focal adhesion occurs within the first two hours following plating so does not coincide with our observed increase in ERK3 levels and thus disappear at the same time as the ERK3 protein level start to increase. It is possible that ERK3 is recruited to the MK5-Src complex at the focal adhesion later in the adhesion response and become tyrosine phosphorylated by Src^48^ and this give rise to both increased ERK3 stability and inability to activate MK5. Thus in our cells ERK3 is localized to areas of the cell that can deliver increased contractility.

**Figure 9.**
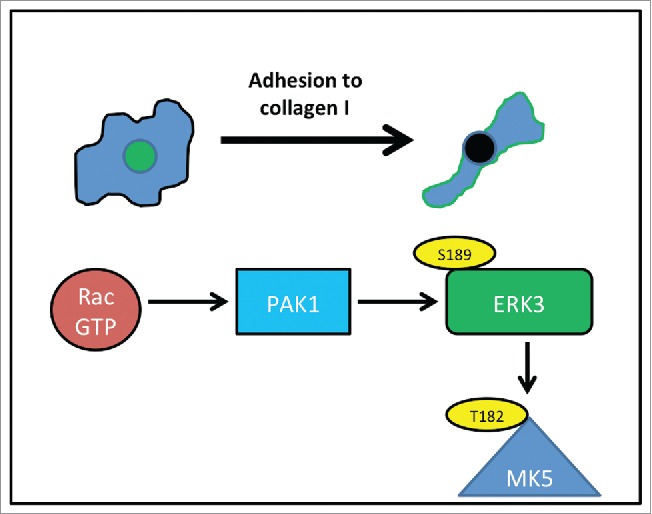
ERK3 activity during nascent adhesion. As cells adhere to collagen I, Rac is activated, activated Rac binds to PAK1 and induces phosphorylation of ERK3 at serine189 – phosphorylation leads to peripheral localization of ERK3 (green). Phosphorylation also leads to interaction with MK5 although this may not be part of the ERK3 morphological response. Localization of ERK3 at the cell periphery is required for the morphological changes that occur during nascent adhesion.

We have observed that overexpression of ERK3 induces cell rounding and changes in the actin cytoskeleton configuration. While studies have linked the ERK3 substrate, MK5 to actin cytoskeletal dynamics.^31,32^ this is the first report of a direct influence by ERK3. Moreover, our studies suggest that this function is not mediated via phosphorylation of and activation of MK5. Although we cannot rule out the possibility that ERK3 functions to stabilize MK5 and allow downstream activation of MK5 via interaction with ERK4 and/or p38.^47,49^ How ERK3 could deliver a change is cellular contractility is currently unknown. MK5 activity has been linked to regulation of a Rho family GEF,^50^ but not specifically to contractility while there are no other known substrates for ERK3. The Rho-ROCK signaling pathway is known to be involved in actomyosin contractility and associated with rounded-amoeboid cells^9,10^ thus it would be of interest to explore whether there is any functional relationship between ERK3 and the Rho pathway. In contrast, PAKs are a family of proteins with strong links to regulation of the actin cytoskeleton.^21^ Indeed, PAKs are downstream effectors of Rac1 which is known to be activated when cells are plated on an extracellular matrix.^17,51^ It is interesting to note that PAKs are known to drive the association of MK5 and ERK3 via serine189 phosphorylation.^30^ It is therefore conceivable that Rac induced phosphorylation of ERK3 via PAK could be a signaling event during cell shapes changes following adhesion formation (**Fig. 9**). Indeed, PAK1 mediated phosphorylation is known to re-localize ERK3 to the cytoplasm and we now find that phosphorylation at serine 189 is required for peripheral localization following nascent adhesion. ERK3KD cells were unable to deliver a reduction in cell spread area following plating, which supports a role for ERK3 in mediating this morphological event. Interestingly the ERK3KD cells ultimately exhibited increased elongation compared to controls cells suggesting that under normal physiological conditions ERK3 localized at the cell periphery is required to prevent hyper-elongation.

We observed that overexpression of ERK3 could induce cell rounding and also increase mean migration speed of the MDA-MB-231 cells. This is the first recorded observation of a direct effect of ERK3 expression on cell migration. Cancer cells have been reported to adopt differential modes of migration (mesenchymal and amoeboid types)^52^ and it has been observed that the less adherent rounded/amoeboid like cancer cell exhibits an elevated cell migration speed.^53^ It is therefore not surprising that ERK3 induced round cells displayed an increased mean migration speed. Our data suggest that ERK3 mediated rounding is not dependent on kinase activity, and thus the increase in cell migration speed is also likely to be kinase independent. We cannot rule out however, that ERK3 performs an MK5 stability function, indeed, MK5 activity has been associated with cell motility,^32,33,54^ where depletion of MK5 impaired chemotaxis. Indeed, the function of ERK3 downstream of PAK activation maybe to localize MK5 to the cell periphery. Our data led us to expect that depletion of ERK3 expression might inhibit cell migration, however we found that ERK3KD cells exhibited normal migration speeds. Although unexpected it is perhaps not that surprising. We have identified a specific role for ERK3 during cell plating whereby ERK3 is required to orchestrate specific cell morphology changes. Such morphology changes are not necessarily required during random migration. Furthermore, we have observed that ERK3 induced rounded cells display an increase in cell migration speed but this does not necessarily mean that a more mesenchymal shaped cell, as observed for ERK3KD, would exhibit a cell migration speed lower then control. Indeed, elongated mesenchymal cells are able to efficiently migrate.^9,10^ It may be that ERK3KD cells would exhibit migration defects in a more constrained environment where increased cellular contractility is required, such a migration through a 3D matrix.^55-57^ Complementary to our random migration studies we also observed that ERK3 overexpression was able to drive MCF-7 cell scattering, a cellular response known to be mediated by PAK activation,^58^ suggesting that ERK3 mediated cell morphological changes are not restricted to one cell type.

## Conclusion

We have clearly demonstrated an important specific role for ERK3 in mediating cell morphological changes. Furthermore, we were able to accurately localize endogenous ERK3 to the cell periphery. We found that both cells overexpressing ERK3 and ERK3 knockdown cells exhibited significant changes in spread area and elongation ratio compared to control cells. Moreover, in two different migration assays overexpression of ERK3 increased cell migration potential. ERK3 mediated cell morphology changes were found to be independent of MK5 activation. We speculate from our results and previous work that activation of RAC during nascent cell adhesion and subsequent cell migration promotes the localization of ERK3 at the cell periphery via PAK1 phosphorylation. ERK3 localized at the cell periphery is able to mediate the cell morphological changes associated with recently plated cells and also drive cell migration speed via increased cell rounding. These ERK3 functions may be the result of interaction with MK5 or via an as yet undefined molecular pathway. Thus we have identified a novel function for ERK3 that may have important consequences for therapeutic intervention of cancer cell invasion.

## Materials and Methods

### Immunoblotting and antibodies

MDA-MB-231 and MCF-7 cells were lysed for 10 minutes in lysis buffer (0.5% NP-40, 30 mM sodium pyrophosphate, 50 mM Tris-HCl pH 7.6, 150 mM NaCl, 0.1 mM EDTA, 50 mM NaF, 1 mM Na_3_VO_4_, 1 mM PMSF, 10 μg/ml leupeptin, 1mM DTT and 1 μg/ml aprotinin) and were cleared by centrifugation at 13,000 g for 10 minutes at 4°C. Equal amounts of protein were analyzed by 7.5% SDS-polyacrylamide gels then transferred into nitrocellulose membranes (Schleicher and Schell). Western blotting was performed by blocking nitrocellulose membranes with 5% skim milk in 1XTBST buffer for 1 hour, followed by overnight incubation with primary antibody at 4°C and 1 hour incubation with the appropriate secondary antibody at room temperature. Blots were developed by enhanced chemiluminescence (ECL, Amersham Pharmacia). The MAPK6 (ERK3) monoclonal antibody (M02), clone (4C11) was purchased from Abnova. The 9E10 c-Myc (sc-40) was purchased from Santa Cruz Biotechnology. Monoclonal anti-Flag (cat#1804), clone (M2) was purchased from Sigma Aldrich. Alexa Fluor 488 goat anti-mouse IgG (A-11001) and Cy5 goat anti-mouse IgG (A10524) were purchased from (Invitrogen). Both polyclonal goat anti-mouse and anti-rabbit immunoglobulins HRP secondary antibodies were purchased from Dako. p44/42 MAPK (ERK1/2) antibody was purchased from Cell Signaling and PRAK (MK5) (A-7) monoclonal antibody (sc-46667) was purchased from Santa Cruz.

### DNA constructs

The following construction pSG5ERK3-Myc (MYC-ERK3), pSGERK3D171A-Myc (MYC-ERK3D171A) and pSGERK3S189A-Myc (MYC-ERK3S189A) had been described previously.^30,45^ EGFP-ERK3 (GFP-ERK3) was generated by cloning the EcoRI-SalI fragment from pGBKT7-ERK3.^59^ in the corresponding sites of pEGFP-C1 (Clontech). The zebrafish ERK3 was amplified from the image clone (IMAGE:9038031) using primers 5′- ggcgaattcatcacagaatggcagagaaatttgaaagc-3´and 5´- gcggatccttacttatcgtcgtcatccttgtaatcatttaaatgcttgaaaatgctgc -3 and subcloned as an *EcoRI-BamHI* fragment into pSG5 vector (Stratagene) generating pSG5-Flag-zfERK3. Note that at the protein level zfERK3 and HuERK3 have 95% amino acid sequence similarity in the kinase domain.

### shRNA

shRNA against ERK3 was cloned in the retroviral vector L193 RRI-GreenattR1ccdBCmRattR2 (kindly provided from Dr D. Micklem, University of Bergen). The procedures are described in Henriksen JR et al. study.^60^ The following sequence was used for ERK3 shRNA (hairpin in lower case): GGCTTTTCATGTATCAGCTTTCaagcttCAAAGCTGATACATGAAAAGCC

The negative control shRNA against Luc was cloned in the same vector and was kindly provided from Dr. C. Einvik, University of Tromsø.

### Viral transduction

Phoenix AMPHO cells were transfected with the retroviral constructs using calcium phosphate precipitation. Forty-eight hours after transfection the supernatant was harvested mixed with 5ug/ml protamine sulfate and used to infect recipient cells. Stable knockdown cell lines were selected from the infected cells by growing them in presence of 1ug/ml puromycin.

### Cell culture and transfection

MDA-MB-231 (American Type Culture Collection (ATCC) HTB-26), MCF-10 (ATCC CRL-10317), MCF-7 (ATCC HTB-22), HeLa (ATCC® CCL^-^2™), 293T/17 [HEK 293T/17] (ATCC® CRL-11268™) and Phoenix AMPHO cells (ATCC CRL-3213) were maintained in Dulbecco's Modified Eagle's Medium from Sigma-Aldrich (D 5796) supplemented with 10% fetal bovine serum, penicillin (100 units/ml), and streptomycin (100 μg/ml). Lipofectamine 2000 (Invitrogen) reagent was used to transfect the MDA-MB-231 and MCF-7 cells according to the manufacturer's instructions.

### Immunofluorescence and image analysis

Cells were seeded at a density of 5×10^4^ cells/ml on collagen I (BD Biosciences) coated coverslips for overnight. To detect the morphological changes in ERK3 transfected cells, cells were transfected with either MYC-ERK3 or GFP-ERK3 for overnight. All cells were subsequently fixed with 4% paraformaldehyde in PBS for 20 minutes at room temperature and then permeabilised with 0.2% Triton X-100 in PBS for 5 minutes. The cells were than blocked with 3% bovine serum albumin in PBS for 30 minutes. Following incubation, cells were washed three times in PBS. For the detection of ERK3, primary antibody was diluted in PBS (1:50) containing 0.5% bovine serum albumin and was incubated for 2 hours at room temperature. The cells were then washed three times in PBS and incubated with the secondary antibody along with Rhodamine phalloidin (Invitrogen) diluted in PBS (1:1000) for 1 hour at room temperature. For cell morphology detection, images were taken using Olympus 1X71. For ERK3 localization and stabilization, images of cells were obtained using a Zeiss LSM510 confocal laser-scanning microscope (Zeiss, Welwyn Garden City, UK), using the accompanying LSM 510 software. Cell spread area (area), elongation ration (circularity) and perimeter were analyzed using ImageJ (NIH) program. Data are presented as mean ± s.e.m. The Student paired t-test was used to compare differences between groups. Statistical significance was accepted for P ≤ 0 .05.

### Time-lapse microscopy

Time-lapse microscope was also used to display a movie were cells had been tracked. MDA-MB-231 cell were seeded on 6-well plates, containing control or experimental cells as described in the figure legends, were placed on the automated stage of a Olympus 1X71 in the presence of 10% CO_2_. Cell images were collected using a Sensicam (PCO Cook) CCD camera, taking a frame either every 3 minutes for 8 hours or 5 minutes for 16 hours from each of the 6 wells using AQM acquisition software (Andor Technology, Belfast, UK). Subsequently, all the acquired time-lapse sequences were displayed as a movie and cells were tracked for the whole of the time-lapse sequence using AQM tracker (Andor Technology, Belfast, UK).

### Cell protrusion activity analysis

Outlines of cells and cell clumps were determined using our own bespoke image processing routines in Wolfram Mathematica 10 (Champaign, IL). These outlines were filled so that cells were represented as white objects on a black background. Consecutive images were combined in to an RGB image such that the first frame constituted the red channel and the next time frame was the green channel. The blue channel was left blank. Thus, red pixels represented regions that had undergone retraction, while green regions represented regions that had undergone protrusion. Regions of overlap (yellow) were converted to gray. This was done for each consecutive pair of images in a temporal stack. Protrusion and retraction indices were calculated on a per image basis by counting the number of red and green pixels and divided by the available cellular perimeters as determined from the detected cellular outlines.

### Reverse transcription quantitative PCR (RT-qPCR)

Total RNA was isolated from cells using the RNeasy Plus kit from Qiagen according to the manufacturer's recommendations. Quantity and purity of the extracted RNA was determined using the NanoDrop spectrophotometer (Thermo Fisher Scientific), and mRNA expression levels were quantified by reverse transcription quantitative PCR (RT-qPCR) performed on a Stratagene MX3000P instrument. Reverse transcription of total RNA was performed using Reverse Transcriptase Core Kit (Eurogentec) with random nonamer primers according to the manufacturer's recommendations.

Primer pairs were purchased from Sigma Life Science. Primer specificities and absence of primer dimers were determined by SYBR green melting curve analysis. cDNA was amplified for 40 cycles in a 25 µl SYBR green PCR mix (Brilliant II SYBR Green QPCR Master Mix, Stratagene) containing 300 nM of each primer. Cycling conditions: 95ºC for 10 minutes, 40 cycles at 95 ºC for 30 seconds, and 60ºC for 1 minute. Duplicate reverse transcriptase reactions were performed for each RNA sample, and duplicate PCR analyses were performed on each cDNA sample. The absence of genomic DNA was confirmed by performing a no reverse transcriptase (NoRT) control, and the absence of contaminations was assessed by including a no template control (NTC). The delta-delta Cq method.^61^ was used to determine the relative amount of target mRNA in the samples normalized against the average expression of the two reference genes *ACTB* and *TFRC*.
