# Supplementary material for: A novel role for atypical MAPK kinase ERK3 in regulating breast cancer cell morphology and migration
Source: Cell Adh Migr. 2015 Nov 20;9(6):483–94. doi: 10.1080/19336918.2015.1112485 (PMC4955959; doi:10.1080/19336918.2015.1112485)
Supplement: Supplemental_Figure_1.zip [file kcam-09-06-1112485-s001.zip › Figure S1 legend.docx]

**Figure S1. (A) ERK3 increasing level up to 9 hours.** HeLa cells were seeded onto collagen I plates and harvested at the following time course 0.5, 1, 2, 4, 6, 8 and 9 hours. The cells were then probed for endogenous ERK3, MK5, ERK2 and Actin. s=cells in suspension. **(B)** **Relative amount of** **ERK3 mRNA in MDA-MB-231 and HEK293 cells were analyzed by RT-qPCR.** MDA-MB-231 and HEK293 cells were seeded onto collagen I coverslips for the following time course 0, 1, 2, 3, 4 and 6 hours. **(C) Overexpressed ERK3 localizes mainly in the nucleus and at the plasma membrane of the cell.** MDA-MB-231 cells were transfected with GFP-ERK3 for 24 hours, fixed and stained with TRITC-phalloidin for F-actin (red). Confocal images were taken. **(D)** **ERK3 localizes mainly in the nucleus and at the plasma membrane of the cell.** MDA-MB-231 cells were fixed after seeding for 24 hours and stained with TRITC-phalloidin to show F-actin (red), Dapi (blue). For ERK3 detection (green), ERK3 monoclonal antibody was used followed by Cy5 anti-mouse. Confocal images were taken. **(E and F)** Control cells were exposed to transfection reagent and processed as per Figure 4 and 5 respectfully; arrows indicate peripheral prominent actin stress fibres. Scale bar: 10μm. **(G) Flag-zfERK3 is functional in MDA-MB-231 cells**. MDA-MB-231 cells were transfected with Flag-zfERK3 for 24 hours (and treated the same as in Figure 4). The cells were fixed and stained with anti-Flag (green), TRITC-phalloidin (red) and DAPI (blue). Scale bar: 10μm. Four representative images from three separate experiments.
