# Supplementary figures and images for: A novel role for atypical MAPK kinase ERK3 in regulating breast cancer cell morphology and migration
Source: Cell Adh Migr. 2015 Nov 20;9(6):483–94. doi: 10.1080/19336918.2015.1112485 (PMC4955959; doi:10.1080/19336918.2015.1112485)

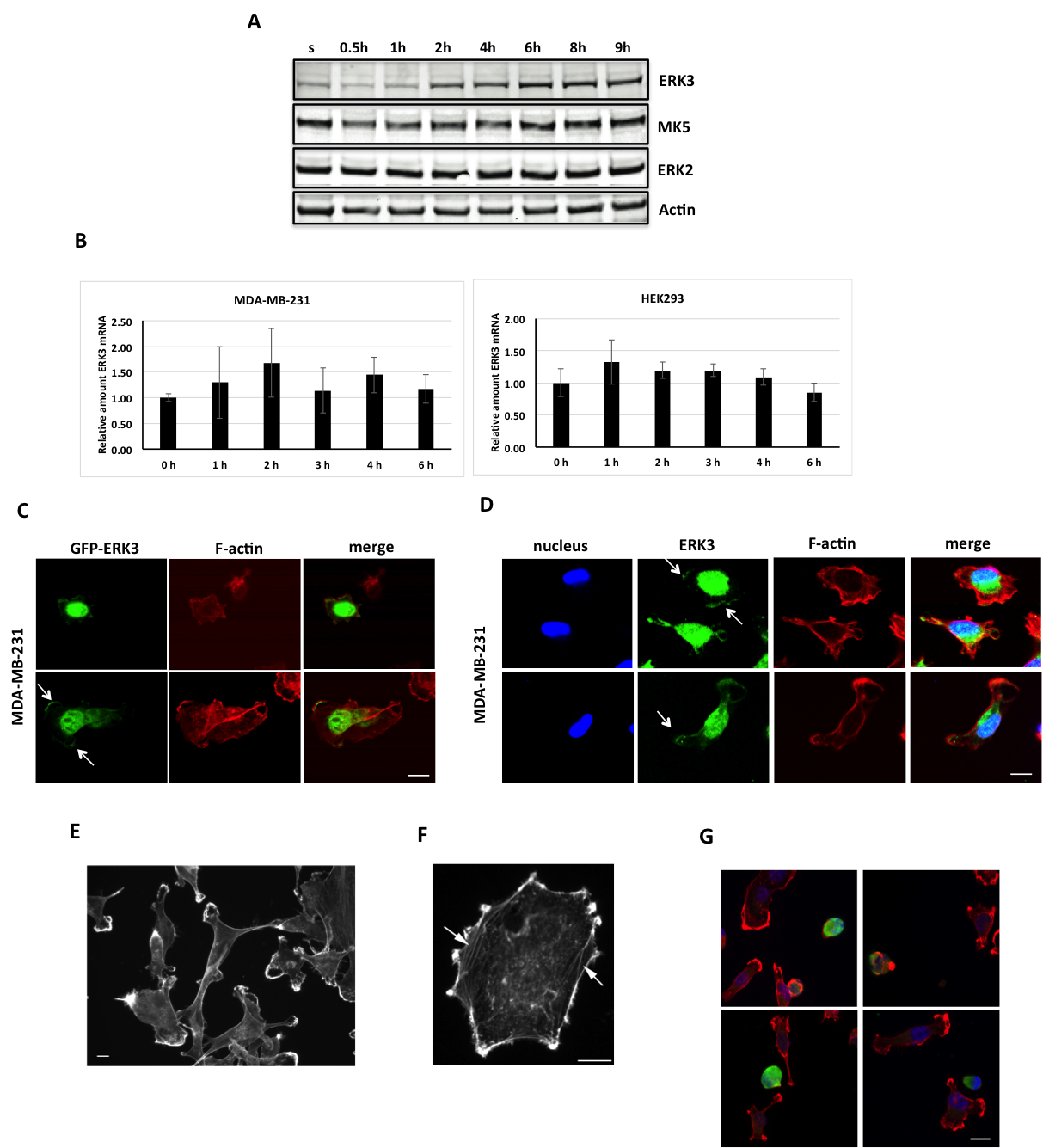

Supplement: Supplemental_Figure_1.zip [file kcam-09-06-1112485-s001.zip › Supplemental Figure 1.tif]
